# Liquid biopsies based on DNA methylation as biomarkers for the detection and prognosis of lung cancer

**DOI:** 10.1186/s13148-022-01337-0

**Published:** 2022-09-24

**Authors:** Peilong Li, Shibiao Liu, Lutao Du, Ghazal Mohseni, Yi Zhang, Chuanxin Wang

**Affiliations:** 1 Department of Clinical Laboratory, The Second Hospital, Cheeloo College of Medicine, Shandong University, Jinan, 250033 Shandong China; 2 Shandong Engineering and Technology Research Center for Tumor Marker Detection, Jinan, Shandong China; 3 Shandong Provincial Clinical Medicine Research Center for Clinical Laboratory, Jinan, Shandong China; 4 Department of Respiratory and Critical Care Medicine, Qilu Hospital of Shandong University, Jinan, Shandong China

**Keywords:** Lung cancer, DNA methylation, Liquid biopsy, Technology, Detection, Prognosis, Biomarker

## Abstract

Lung cancer (LC) is the main cause of cancer-related mortality. Most LC patients are diagnosed in an advanced stage when the symptoms are obvious, and the prognosis is quite poor. Although low-dose computed tomography (LDCT) is a routine clinical examination for early detection of LC, the false-positive rate is over 90%. As one of the intensely studied epigenetic modifications, DNA methylation plays a key role in various diseases, including cancer and other diseases. Hypermethylation in tumor suppressor genes or hypomethylation in oncogenes is an important event in tumorigenesis. Remarkably, DNA methylation usually occurs in the very early stage of malignant tumors. Thus, DNA methylation analysis may provide some useful information about the early detection of LC. In recent years, liquid biopsy has developed rapidly. Liquid biopsy can detect and monitor both primary and metastatic malignant tumors and can reflect tumor heterogeneity. Moreover, it is a minimally invasive procedure, and it causes less pain for patients. This review summarized various liquid biopsies based on DNA methylation for LC. At first, we briefly discussed some emerging technologies for DNA methylation analysis. Subsequently, we outlined cell-free DNA (cfDNA), sputum, bronchoalveolar lavage fluid, bronchial aspirates, and bronchial washings DNA methylation-based liquid biopsy for the early detection of LC. Finally, the prognostic value of DNA methylation in cfDNA and sputum and the diagnostic value of other DNA methylation-based liquid biopsies for LC were also analyzed.

## Background

Lung cancer (LC) is ranking as the most common malignant tumor and the predominant cause of mortality among cancers worldwide [1]. Clinically, the early manifestations of LC are atypical, and the disease has already entered an advanced stage when the symptoms are obvious. Consequently, a lot of patients have been diagnosed as an advanced stage at the first visit to hospital, and the 5-year overall survival rate of an advanced stage is very low [2]. The most commonly used approach for LC screening is low-dose computed tomography (LDCT) [3]. However, due to the high sensitivity of LDCT, sometimes some benign lesions are misdiagnosed as malignant tumors. Therefore, there is an urgent need to increase the early detection rate of LC through novel biomarkers and improve patients’ prognosis.

**Fig. 1:**
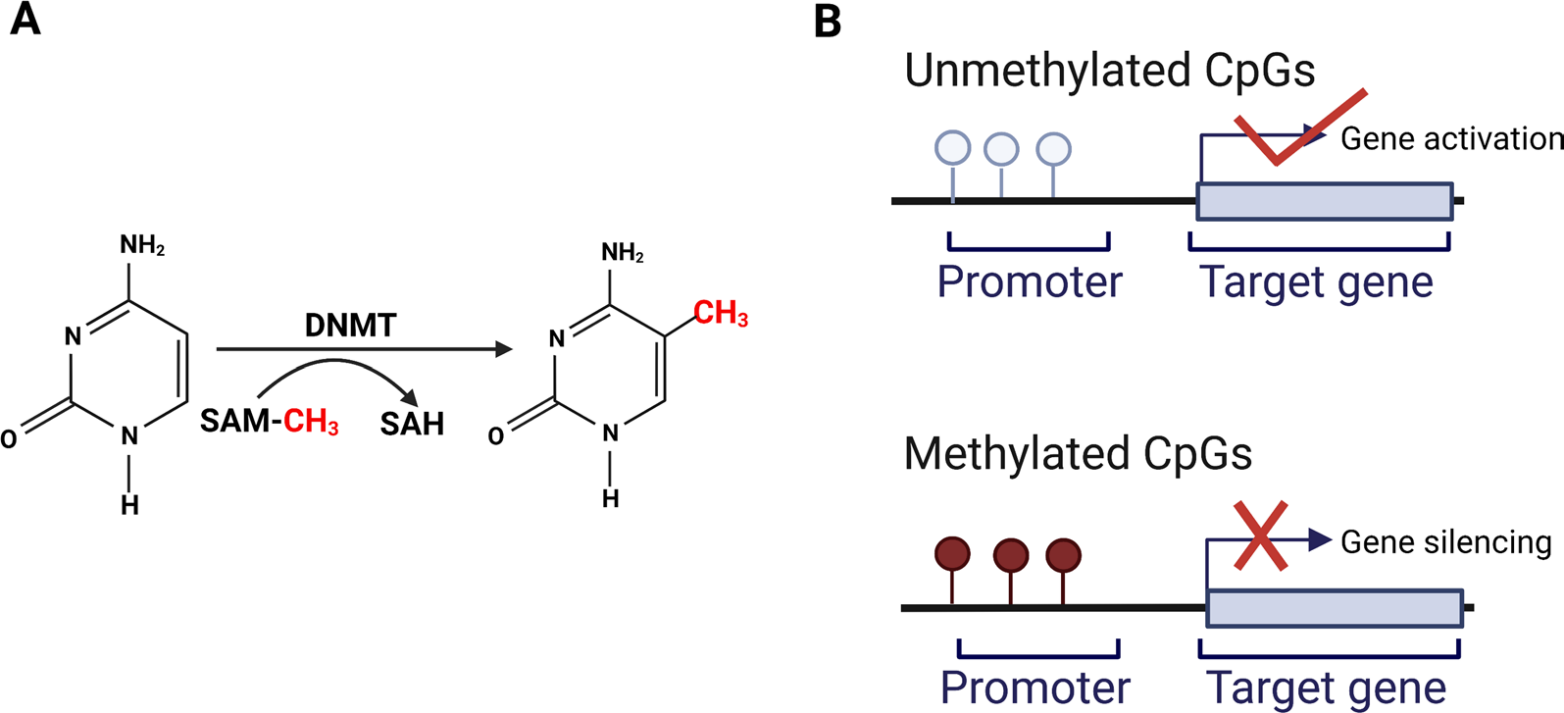
Formation of DNA methylation and its regulation of gene expression. **A** Process of DNA methylation. DNA methylation usually happens in CpG islands by adding a methyl group, provided by S-adenosylmethionine (SAM), to the carbon-5 position of a cytosine. This process is catalyzed by DNA methyltransferases (DNMTs). **B** DNA methylation of gene promoters. Unmethylated CpGs result in gene activation. Methylated CpGs lead to gene silencing

Epigenetics is the study of heritable changes that do not involve any change in DNA sequences. Epigenetic mechanisms that are responsible of these changes include DNA methylation, histone modifications, and non-coding RNAs regulation. Unlike genetic changes, epigenetic alterations can be reversible. Abnormalities in epigenetics may lead to cancer and autoimmune diseases [4]. DNA methylation, one of the widely studied epigenetic modifications in humans, occurs by adding a methyl group to the 5-position carbon of a cytosine, which is catalyzed by DNA methyltransferases (DNMTs). DNA methyltransferase’s function is to build and maintain the methylation patterns [5, 6] (Fig. 1A). Usually, this process occurs in regions with a large number of CpG dinucleotides called CpG islands. DNA methylation is crucial for gene expression (Fig. 1B). Besides, it is also involved in various biological processes, such as maintenance of chromatin architecture, X-chromosome inactivation, and genomic imprinting [7]. Thus, dysregulation of DNA methylation may contribute to various diseases, including cancer and other diseases. DNA methylation can change chromatin structure, stimulate methylated binding-protein (MBP) to bind to transcription inhibitors, and prevent transcription factors (TFs) from binding to DNA sequences and consequently affecting gene transcription [8]. Previous studies have demonstrated that hypermethylation in promoters represses gene transcription. In contrast, hypomethylation in promoters promotes gene transcription. As for the body of the gene, DNA methylation in gene body may promote transcription of the gene. One explanation is that DNA methylation in gene body may influence activities of repetitive DNAs in the transcriptional unit [9]. Hypermethylation in tumor suppressor genes or hypomethylation in oncogenes is associated with tumorigenesis [6, 10]. Intriguingly, aberrant DNA methylation usually occurs at an early stage of tumorigenesis. Besides, aberrant DNA methylation is related to cancer progression. For example, DNA methylation alterations in *TBC1D16* and *EBF3* play a vital role in the progression and metastasis of colorectal cancer, prostate cancer, and melanoma [11]. In addition, DNA methylation is reversible and may help monitor the therapeutic effects. Therefore, DNA methylation can provide a new strategy for early detection of LC and improve its prognosis.

**Fig. 2:**
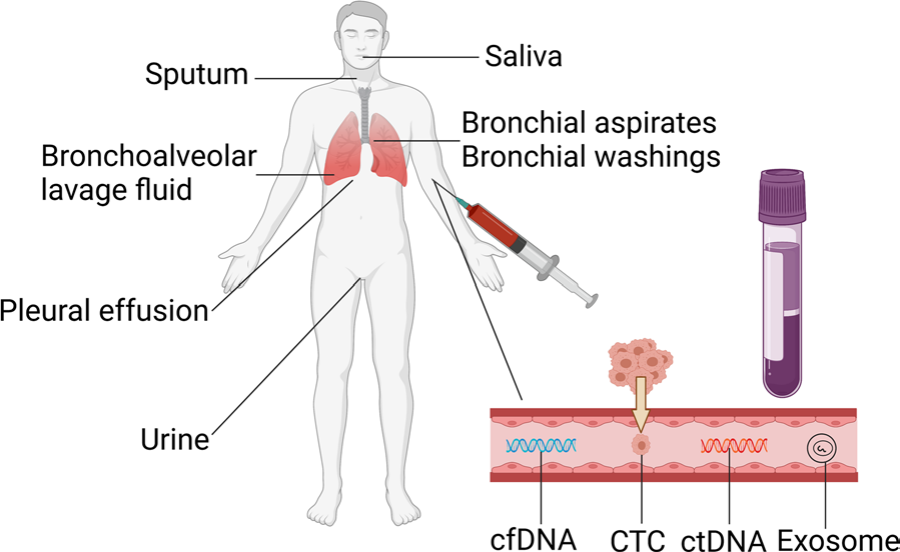
Multiple liquid biopsy methods. Clinical information can be obtained from liquid biopsies. Among them, the main objects of liquid biopsy are cell-free DNA (cfDNA), circulating tumor cells (CTCs), and exosomes. Liquid biopsy is minimally invasive. Particularly, the collection of sputum, saliva, and urine is completely noninvasive. Hence, liquid biopsy has a promising clinical application

Nowadays, liquid biopsy has received enormous attention as it has emerged as a minimally invasive approach for detecting cancer at an early stage [12]. Liquid biopsies mainly include cell-free DNA (cfDNA), circulating tumor cells (CTCs) and exosomes. In addition to the above-mentioned, sputum, urine, saliva, bronchoalveolar lavage fluid, bronchial aspirates, bronchial washings, and pleural effusions are also the forms of liquid biopsies [13–16] (Fig. 2). Importantly, one of the advantages of liquid biopsy is that it can continuously monitor the evolution of both primary and metastatic malignant tumors and detect their recurrences [17]. Besides, it is minimally invasive and cost-effective, which makes it more acceptable for patients [18]. Compared to conventional LC-related tumor markers, the sensitivity and specificity of liquid biopsy are higher. LDCT has disadvantages of a high false-positive rate and more or less radiation exposure, while liquid biopsy can overcome these problems. Aspiration biopsy of a suspicious lung lesion is traumatic and some patients have poor compliance. On the contrary, liquid biopsy has attracted a lot of attention and several reports have revealed the clinical application of liquid biopsy in LC [19]. For instance, detection of plasma-derived *EGFR* mutation of advanced or metastatic non-small cell lung adenocarcinoma (NSCLC) patients is helpful for the treatment with *EGFR* tyrosine kinase inhibitors (TKIs) [20]. Another study found that plasma circulating tumor DNA (ctDNA) frequencies can be a powerful tool for monitoring the treatment response of NSCLC patients [21]. As DNA methylation occurs at an early stage of malignant tumors, it is expected to be a novel biomarker, and can be a potential field in liquid biopsy.

In this review, we summarized multiple liquid biopsy methods based on DNA methylation for LC. Firstly, we briefly reviewed some emerging technologies for DNA methylation analysis. Next, we focused on DNA methylation of cfDNA, sputum, bronchoalveolar lavage fluid, bronchial aspirates, and bronchial washings for early detection of LC. Also, the prognostic value of DNA methylation in cfDNA and sputum and the diagnostic value of other DNA methylation-based liquid biopsies for LC were discussed.

## Technologies for DNA methylation analysis

Considering the critical role of DNA methylation in tumorigenesis and cancer screening, how to detect DNA methylation status accurately and quickly is particularly important. Here, we reviewed some technologies for DNA methylation analysis. Particularly, we focused on some emerging technologies.

### Technologies for genome-wide DNA methylation analysis

As for technologies for genome-wide DNA methylation analysis, it includes two aspects: technologies for determination of global DNA methylation analysis and technologies for whole-genome DNA methylation profiling analysis. Global DNA methylation analysis includes enzyme-linked immunosorbent assay (ELISA) and luminometric methylation assay (LUMA). ELISA is a rapid, convenient, sensitive, and inexpensive method for the quantitative measurement of 5mC. However, the disadvantages of ELISA are cross-reactivity and low specificity. LUMA is a good approach to analyzing DNA methylation [22]. The main principle of LUMA is based on DNA digestion, which HpaII or MspI catalyzes. The sensitivity of LUMA is really high. The whole-genome DNA methylation profiling analysis includes methylation BeadChip and next-generation sequencing (NGS). Human methylation 850 K BeadChip (MethylationEPIC BeadChip), developed based on human methylation 450 K BeadChip, is a method based on probe hybridization. It can analyze more than 850,000 CpG sites, including genome coverage of enhancers, transcription factor binding sites, open chromatin, and miRNA promoter regions. It is sensitive, time-effective, and user-friendly. However, a main limitation of MethylationEPIC BeadChip is that it only detects the previously determined regions where the probes can be designed, and the cost is high. Agilent’s Human DNA Methylation Microarrays are other platforms to quantify 5mC distribution in genomes. Briefly, 5mC-specific monoclonal antibodies are used to pull down methylated DNA fragments. Then, microarray probes hybridize with methylated regions. A disadvantage of microarrays is that they only cover a part of CpG sites, and some CpG sites may not be detected [23, 24]. Methylated DNA immunoprecipitation (MeDIP) is a technology based on the IP principle to detect DNA methylation levels. A monoclonal antibody against 5mC is used to bind to methylated regions of DNA samples. Then, NGS (MeDIP-Seq) or DNA microarrays (MeDIP-Chip) analyzes the methylated DNA fragments. This technology has been applied to some cancers, such as breast cancer and ovarian cancer. Methyl-CpG binding domain (MBD) protein capture (MethylCap) assay analyzes genome-wide DNA methylation by MBD-specific antibody. The principle and procedure are similar to MeDIP. However, MethylCap is more sensitive than MeDIP in terms of detecting CpG islands. MethylCap has been applied to identify methylation-relevant biomarkers for lung cancer [25], bladder cancer [26], and leukemia [27]. Enzymatic methyl-seq is based on two sets of enzymatic reactions, mainly detecting 5mC and 5hmC. In the first step, 5mC and 5hmC are converted to products by TET2 and T4-BGT. APOBEC3A cannot deaminate these products. In the second step, APOBEC3A converts unmodified cytosines to uracils through deamination. Therefore, these three enzymes (TET2, T4-BGT, and APOBEC3A) can identify 5mC and 5hmC [28]. HpaII tiny fragment enrichment by ligation-mediated PCR (HELP) is another enzymatic-based sequencing technology. At first, MspI (resistant to 5mC) and HpaII (sensitive to 5mC) digest genomic DNA, respectively. Then, the restriction fragments are labeled with two specific fluorochromes and are amplified by ligation-mediated PCR. Finally, the products are directly sequenced (HELP-Seq) or hybridized to a microarray (HELP-Chip). This method is sensitive, and it can detect genomic regions, regardless of CpG density [24]. Whole-genome bisulfite sequencing (WGBS) is a typical type of NGS, and it analyzes each CpG site of bisulfite-converted DNA. The advantages of WGBS are high accuracy and good repeatability. Furthermore, WGBS is a sensitive method, and it only needs ~ 30 ng of DNA, even 150 pg in some cases. However, this technique is expensive and the operation is complex. Reduced representation bisulfite sequencing (RRBS) is an adaptation of WGBS. Compared to WGBS, the cost of RRBS is lower and the throughput is higher. Nevertheless, it needs more DNA than WGBS, and its sensitivity is slightly lower than WGBS [29, 30].

### Technologies for locus-specific DNA methylation analysis

It is sometimes necessary to explore the DNA methylation status of some specific CpG sites. Technologies for locus-specific DNA methylation analysis are performed to solve this problem. Pyrosequencing and PCR are two major categories of technologies for locus-specific DNA methylation analysis. Pyrosequencing provides real-time quantitative information on bisulfite-converted DNA. Briefly, PCR is subjected to amplifying DNA sequencing of interest. Among them, one primer is biotinylated. Pyrosequencing primer is performed to react with DNA template [30]. Although pyrosequencing is a highly quantitative method, sequencing products is a time-taking process, which may reduce the accuracy. For PCR, methylation-specific PCR (MSP) is also used to detect the level of DNA methylation. MSP contains two primers: One binds to methylated CpG sites, and the other is to bind to unmethylated CpG sites. Moreover, MethyLight combines the properties of MSP and TaqMan probe. For sensitivity, MethyLight is ten times more sensitive than MSP. Besides, MethyLight reduced the occurrence of non-specific binding events [31]. Digital PCR (dPCR) is performed by partition method that detects every single well. The DNA sample is divided into many reaction wells. Each well contains or does not contain the target molecule, representing positive or negative result. Droplet digital PCR (ddPCR) is based on a similar principle to dPCR but a bigger scale. The DNA sample can be divided into 20,000 droplets. The result of each droplet is positive or negative. DdPCR is a very sensitive method, and it is 25 times more sensitive than MethyLight [30].

### Newly developed technologies for DNA methylation analysis

In recent years, some new technologies for DNA methylation analysis have developed fast and are gradually being used in cancer screening. Here, we discussed the following two main categories of newly developed technologies: mass spectrometry for DNA methylation analysis and biosensors for DNA methylation analysis.

### Mass spectrometry for DNA methylation analysis

Mass spectrometry is a precise technology for global DNA methylation detection. Usually, nano–ultra-HPLC is coupled to multiple reaction monitoring in a mass spectrometry to achieve the exact quantification of DNA [32]. Mass spectrometry for global DNA methylation analysis is a highly sensitive and precise method with good reproducibility. In addition, this assay runs through the whole genome and it takes no account of sites or sequences. However, mass spectrometry is expensive and the operation is complex [32, 33].

### Biosensors for DNA methylation analysis

#### Optical biosensors

The basic principle of the optical biosensor is that when a biorecognition layer captures the target analyte, the optical biosensor can detect the produced light. Colorimetry and fluorescence are two major detection methods of the optical biosensor. Colorimetric optical biosensors are represented by color changes, making them visible to naked eyes. Compared to colorimetric optical biosensors, the sensitivity of fluorescent ones is greatly higher due to their fluorescence detection [23]. Optical biosensors have advantages, such as high sensitivity, specificity, and repeatability. This approach has been successful in the detection of tumor markers in clinical samples.

#### Electrochemical biosensors

Electrochemical biosensors detect electrical parameter signals. Based on the differences in electrical parameters, electrochemical biosensors can be divided into three major categories: potentiometric biosensors, amperometric biosensors, and impedimetric biosensors [23]. They detect potential, electric current, and resistance, respectively. Usually, electrochemical biosensors contain a working electrode and reference electrode. The working electrode’s function is to detect the analytes, representing the generated response as electrical signals. Like optical biosensors, sensitivity and specificity are very high. Besides, the cost is low and the detection requires a short time. However, a big issue is that the clinical application of electrochemical biosensors is not common until now, which needs to be resolved soon.

The development of detection technology has provided basis for liquid biopsy. Nowadays, liquid biopsy has received enormous attention as it can serve as a minimally invasive approach for early detection of cancer [12]. Liquid biopsies mainly include cfDNA, CTCs, and exosomes. Also, sputum, bronchoalveolar lavage fluid, bronchial aspirates, bronchial washings, urine, pleural effusions, and saliva are the forms of liquid biopsies [13–16]. In the following part, we will introduce different types of DNA methylation-based liquid biopsies for the detection and prognosis of LC.

## CfDNA methylation-based liquid biopsy for LC

CfDNA is released into the bloodstream from both healthy and tumor cells. In patients with malignant tumors, circulating tumor DNA (ctDNA), which is derived from primary or metastatic tumor cells mainly due to necrosis or apoptosis, is a type of cfDNA [34]. Specimen type selection is an important step before analysis, and it is necessary to compare serum-derived and plasma-derived cfDNA. In general, the concentration of serum-derived cfDNA is higher than plasma-derived one. However, ctDNA is hard to be detected in serum, and the amount of some ctDNA fractions in plasma is more than in serum, such as KRAS-mutated fraction [35]. Besides, plasma ctDNA is more stable than that in serum [36, 37]. Therefore, the recent studies have focused on plasma-derived ctDNA methylation status of LC patients. CtDNA carries tumor-specific genetic and epigenetic information, and several studies have reported the role of ctDNA in managing LC [38–40]. Tumor cell DNA differs from healthy cell DNA in some aspects, including DNA methylation. Moreover, DNA methylation status in cfDNA resembles those of cancer tissues, and aberrant DNA methylation is an important event in the early stage of cancer [10]. Although sometimes the fragment of ctDNA is small or the concentration of ctDNA is low, advanced technology has been applied to ctDNA detection [41]. Thus, cfDNA methylation can be a potent biomarker for LC.

### The value of single-gene methylation-based liquid biopsy in LC detection

Up to now, several studies have demonstrated that aberrant ctDNA methylation could be a potential biomarker for early diagnosis and screening of LC (Table 1). For example, Kneip et al. reported that *SHOX2* methylation in plasma had a sensitivity of 60% and a specificity of 90% to differentiate LC patients from healthy controls [42]. Ponomaryova et al. illustrated that plasma-derived *RASSF1A* and *RARB* methylation levels could serve as biomarkers for early detection of LC with a sensitivity of 87% and a specificity of 75% [43]. In 2019, Xu W et al. revealed that the methylation levels of *B3GAT2, BCAR1, HOPX, HOXD11, MIR1203, MYL9, SLC9A3R2, SYT5, VTRNA1-3,* and *HLF* between LC patients and healthy controls were significantly different, suggesting that these ten genes could serve as diagnostic biomarkers for LC [44]. The involvement of the above 10 genes in LC was summarized in Table 2.

**Table 1:** CfDNA methylation-based liquid biopsy for LC

Purpose	Genes	Cases	Controls	Sensitivity/specificity	Samples	Methods	References
Diagnosis	*SHOX2*	208	175	60.0/90.0%	Plasma	qPCR	[42]
Diagnosis	*RASSF1A/RARB*	60	32	87.0/75.0%	Plasma	qMSP	[43]
Diagnosis	*B3GAT2/BCAR1/HOPX/HOXD11/MIR1203/* *MYL9/SLC9A3R2/SYT5/VTRNA1-3/HLF*	41	39	–/–	Plasma	MeDIP-seq and qPCR	[44]
Diagnosis	*DCLK1*	65	95	49.2%/91.6%	Plasma	qMSP	[45]
Diagnosis	*CDH1/NISCH*	40	30	–/–	Plasma	MSP	[46]
Diagnosis	*CDO1/HOXA9/AJAP1/PTGDR/UNCX/* *MARCH11*	83	42	72.1%/71.4%	Serum	MSP and qMSP	[47]
Diagnosis	*RTEL1/PCDHGB6*	70	80	62.9%/90%	Plasma	qMSP	[48]
Diagnosis	*MIR129-2/LINC01158/CCDC181/PRKCB/* *TBR1/ZNF781/MARCH11/VWC2/SLC9A3/* *HOXA7*	18	47	83.0%/95.0%	Plasma	Two-set qPCR	[49]
Prognosis	*DCLK1*	37	0	–/–	Plasma	qMSP	[45]
Prognosis	*BRMS1*	122	24	–/–	Plasma	qMSP	[50]
Prognosis	*KMT2C*	139	60	–/–	Plasma	qMSP	[51]
Prognosis	*SOX17*	122	49	–/–	Plasma	qMSP	[40]

**Table 2:** The involvement of the single gene in LC

Gene	The involvement of the gene in LC	References
*B3GAT2*	–	–
*BCAR1*	*B3GAT2* promotes the formation and immune evasion of CTCs in LUAD by triggering EMT via RAC1 signaling and up-regulating CD274 expression by shuttling BRD4-S into the nucleus	[52]
*HOPX*	ΜicroRNA-421 promotes the progression of NSCLC by targeting *HOPX* and regulating the Wnt/β-catenin signaling pathway	[53]
*HOXD11*	–	–
*MIR1203*	*MIR1203* level is negatively regulated by LINC00632 to accelerate lymph node metastasis and distant metastasis of NSCLC	[54]
*MYL9*	Low *MYL9* expression may be relevant to the development and metastasis of NSCLC	[55]
*SLC9A3R2*	–	–
*SYT5*	–	–
*VTRNA1-3*	–	–
*HLF*	Genetic deletion and methylation lead to down expression of *HLF*, thus accelerating anaerobic metabolism to promote the growth of NSCLC cells by activating NF-κB/p65 signaling through disrupting translocation of *PPARα* and *PPARγ*	[56]

Gene promoter methylation, which has been studied intensely, plays a leading role in regulating its target gene expression. Furthermore, it has been found that the levels of these epigenetic alterations were very low in healthy people, which reflected its vital role in tumorigenesis [57]. Thus, many studies have focused on gene promoter region methylation status. A study comprised 65 LC patients and 95 healthy controls, of which 32 LC patients (49.2% of the studied group) and 8 healthy controls (8.4% of the control group) showed *DCLK1* promoter methylation. Statistical analysis showed that the test’s sensitivity was 42.9% and the test’s specificity was 91.6%. Further analysis suggested that there was no significant correction between cigarette smoking and *DCLK1* promoter methylation [45]. In 2019, Krishnamurthy et al. revealed that the level of *CDH1* promoter methylation was significantly higher in LC patients than in healthy controls, and the level of *NISCH* promoter methylation was significantly higher in LC patients and non-cancerous smokers than non-smoker controls. Moreover, no significant association was found between the methylation level of *CDH1* or *NISCH* and clinicopathologic variables, such as tumor size, histopathological grading stage, lymph node involvement, and distant metastasis status [46]. All the studies mentioned above suggested that gene promoter methylation has a wide prospect of LC screening.

### Multiple genes panel of ctDNA methylation in liquid biopsy for LC screening

Undoubtedly, ctDNA methylation alterations could differentiate LC patients from healthy individuals. Nevertheless, the diagnostic power of a single methylation site was limited. Interestingly, some researchers tried to establish diagnostic panel based on several different methylation sites for the early detection for LC (Table 1). Ooki et al. used a panel of 6 genes (*CDO1, HOXA9, AJAP1, PTGDR, UNCX,* and *MARCH11*) as a powerful biomarker to distinguish between LC patients and healthy controls, which had a sensitivity of over 90% for diagnosing of stage IA NSCLC [47]. In another study, 70 LC patients and 80 healthy controls were enrolled, of whom 10 ml of peripheral blood was collected. Plasma cfDNA was used to analyze *RTEL1* and *PCDHGB6* promoter methylation status. The sensitivity and specificity of *RTEL1* methylation analysis were 51.4% and 91.2%. For *PCDHGB6* methylation assessment, the sensitivity and specificity were 41.4% and 98.7%. Based on the two genes above, the panel improved diagnostic power with an AUC of 0.75, a sensitivity of 62.9%, and a specificity of 90.0%. The panel showed an AUC of 0.752 with a sensitivity of 64.6% and specificity of 88.4% for operable stages of NSCLC (I-III A) [48]. Vrba L et al. used a biomarker set comprising *MIR129-2, LINC01158, CCDC181, PRKCB, TBR1, ZNF781, MARCH11, VWC2, SLC9A3*, and *HOXA7*, to evaluate its power for detection of NSCLC. The cohort contained 18 NSCLC cases and 47 healthy controls, and ROC analysis showed that the AUC was 0.956, sensitivity was 83%, and specificity was 95%. Additionally, they found a positive relationship between NSCLC stage and methylation levels of the ten genes [49].

### CfDNA methylation-based liquid biopsy for the prognosis of LC

Besides the diagnostic value of DNA methylation, many studies have illustrated gene methylation’s function in the prediction and prognosis of LC (Table 1). In 2015, Powrozek T et al. found that LC patients who presented *DCLK1* promoter region one or region two or both methylated had shorter survival than those without *DCLK1* promoter methylation [45]. Another study focused on the relationship between *BRMS1* promoter methylation and the prognosis of LC patients, suggesting that patients with *BRMS1* promoter methylation had a significantly lower overall survival (OS) time and progression-free survival (PFS) time than those without *BRMS1* promoter methylation [50]. Mastoraki S et al. studied the methylation status of *KMT2C* promoters of operable and metastatic NSCLC patients. They found that compared to patients without *KMT2C* promoter methylation, promoter methylation-positive patients had a lower DFS time and OS time in operable NSCLC patients and a lower PFS time and OS time in metastatic NSCLC patients [51]. Balgkouranidou I et al. found that *SOX17* promoter methylation level was higher in NSCLC than in healthy controls. Besides, *SOX17* promoter methylation status in plasma-derived ctDNA was related to OS in advanced NSCLC patients. Analysis of *SOX17* promoter methylation status could provide prognostic information for advanced NSCLC patients [40].

## Sputum DNA methylation-based liquid biopsy for LC

Sputum consists of exfoliated respiratory epithelial cells from the lower respiratory tract, which is the most common type of bronchial epithelial cells. Besides, other respiratory epithelial cell types in sputum may contain some useful molecular information. Recently, sputum has gained enormous attention, as it can be obtained easily and noninvasively [58]. Sputum cytological examination can reflect morphological alterations of the lower respiratory epithelial cells. Nevertheless, the sensitivity of sputum cytology to detect LC is very low, especially in the early stage of LC [59]. Previous studies revealed that smokers tend to produce more sputum, and carcinogenesis of NSCLC is the result of an accumulation of molecular changes because of the effects of long-term smoking to respiratory epithelial cells [60]. Tumor cells cannot be detected using sputum cytological examination, while the DNA of tumor cells has already altered [61, 62]. Since the respiratory epithelial cells in the sputum are exfoliated from the lower respiratory tract, sputum DNA examination can reflect the molecular alterations of LC. For instance, DNA hypermethylation status in sputum could be detected in LC patients before clinical diagnosis [63]. Furthermore, the rate of hypermethylation detected in LC patients in sputum was significantly higher than in cytology [64]. Thus, sputum DNA methylation can be a noninvasive and powerful tool for LC screening.

### Sputum DNA methylation-based liquid biopsy for LC detection

As for LC detection, great studies have revealed the value of sputum DNA methylation-based liquid biopsy (Table 3). In 2015, Hubers AJ et al. investigated the DNA methylation status of *FAM19A4, RASSF1A, 3OST2, APC, PHACTR3, CYGB,* and *PRDM14* in the learning cohort (73 LC cases and 86 healthy controls) and validation cohort (159 LC cases and 154 healthy controls). However, the diagnostic power of a single gene was not satisfactory. A panel containing *RASSF1A, 3OST2,* and *PRDM14* was constructed. The sensitivity and specificity of positive DNA hypermethylation of one or more of these three genes were 82.2% and 66.3% in learning cohort. In validation cohort, the sensitivity and specificity were 79.2% and 64.3%. Compared to sputum cytology examination, DNA methylation analysis of sputum is superior. Remarkably, *RASSF1A, 3OST2,* and *PRDM14* were studied widely in LC patients. In lung carcinogenesis, hypermethylation of the *RASSF1* promoter gene is an early and important event. As an isoform of *RASSF1*, methylation of *RASSF1A* may be a sign of increased risk of LC [65]. The *3OST2* promoter methylation was observed in several malignant tumors, including LC [66]. *PRDM14* accelerates NSCLC cell migration in vitro and may be a therapeutic target of metastatic NSCLC [67]. Then in 2016, Hubers AJ et al. established a diagnostic panel based on the above three genes for early detection of NSCLC with an AUC of 0.79, a sensitivity of 82.9%, and a specificity of 76.4%. The diagnostic performance was superior to every single one [59]. Another study analyzed the relationship between *RASSF1A, APC,* and *CYGB* methylation levels and LC. The combined analysis of the three genes showed a sensitivity of 63% and a specificity of 78% to discriminate LC cases from healthy controls in set 1 (98 LC cases and 90 healthy controls) and a sensitivity of 90% and a specificity of 47% in set 2 (60 LC cases and 445 healthy controls), respectively [64]. In 2017, Hulbert A et al. detected methylation levels in the sputum of 6 genes (*SOX17, TAC1, HOXA7, HOXA9, CDO1,* and *ZFP42*) in both LC patients and healthy individuals. Except for *HOXA9* with a low specificity, all of the five remaining genes were statistically significant. As a result, they selected the best three genes (*SOX17, TAC1,* and *HOXA7*) to establish a panel, which achieved an AUC of 0.89, a sensitivity of 93%, and a specificity of 89%. Moreover, sputum samples were better than plasma samples in the aspect of methylation detection in this study. One explanation for this may be that LC cells can enter into airways at an early stage. This is an advantage of the methylation of sputum over plasma in the early detection of LC [68].

**Table 3:** Sputum DNA methylation-based liquid biopsy for LC

Purpose	Genes	Cases	Controls	Sensitivity/specificity	Samples	Methods	References
Diagnosis	*RASSF1A/3OST2/PRDM14*	232	240	82.2/66.3%	Sputum	qMSP	[67]
Diagnosis	*RASSF1A/3OST2/PRDM14*	261	345	82.9/76.4%	Sputum	qMSP	[59]
Diagnosis	*RASSF1A/APC/CYGB*	161	536	63.0/78.0% in set 1 90.0/47.0% in set 2	Sputum	qMSP	[64]
Diagnosis	*SOX17/TAC1/HOXA7*	150	60	93.0/89.0%	Sputum	qMSP	[68]
Assessment	*P16/MGMT/DAPK/RASSF1A/GATA4/* *GATA5/PAX5α/PAX5β*	371	1063	95.0/54.0%	Sputum	MSP	[60]
Assessment	*PAX5β/MGMT/DAPK/Dal-1/PCDH20/Jph3/Kif1a*	64	64	–/–	Sputum	qMSP	[69]
Assessment	*PAX5α/DAPK/SULF2/PAX5β/CXCL14/* *GATA5/Dal-1*	40	90	–/–	Sputum	qMSP	[69]
Assessment	*ZNF549/SNCA/CCNA1*	29	108	–/–	Sputum	MSP	[70]

### The assessment of smokers at risk for developing LC

Smoking is one of LC’s most important environmental risks. Although National Lung Screening Trial (NLST) has reported that LDCT could reduce LC-related mortality by about 20%, some benign nodules may be diagnosed as malignant tumors due to the high sensitivity of LDCT [71]. Thus, it is urgent to explore a powerful tool to identify smokers at high risk of LC receiving LDCT. Several reports have revealed that smoking could influence the initiation and progression of LC accompanied by a series of molecular variations, including DNA methylation [72, 73]. Moreover, sputum samples are noninvasive and easy to be obtained. Therefore, several studies focused on the value of DNA methylation alterations in sputum to select high-risk smokers who need a LDCT scan (Table 3). In 2017, Lissa D et al. initially selected some LDCT eligible smokers, including 371 surgically resected LC patients and 1063 cancer-free smokers. They found that the methylation levels of eight genes (*P16, MGMT, DAPK, RASSF1A, GATA4, GATA5, PAX5α,* and *PAX5β*) in resected LC patients were higher than those in cancer-free smokers. Remarkably, the diagnostic power of the 8-gene panel was better than clinical risk factors (age, sex, and smoking status). Furthermore, the combine analysis of eight genes and clinical risk factors achieved a better prediction accuracy compared to 8-gene panel alone. Finally, the authors selected a cohort of smokers who did not need to receive LDCT scans according to the current USPSTF screening criteria. The panel of eight genes and clinical risk factors could identify smokers in the cohort who were at high risk of LC, suggesting the superiority of the panel to identify high-risk smokers over the current USPSTF screening criteria [60]. Another report, published by Leng S et al., established a logistic regression modeling of gene methylation to select the high-risk smokers to receive LDCT. The study comprised Colorado cohort and New Mexico cohort. These panels contained *PAX5β, MGMT, DAPK, Dal-1, PCDH20, Jph3,* and *Kif1*a for Colorado and *PAX5α, DAPK, SULF2, PAX5β, CXCL14, GATA5,* and *Dal-1* for New Mexico. The results showed that the discrimination accuracy was 71% for Colorado and 77% for New Mexico, respectively [69]. In another study, Tessema M et al. estimated the promoter methylation levels of three genes (*ZNF549, SNCA,* and *CCNA1*) in sputum from smokers. In cancer-free smokers, the methylation levels of *ZNF549* and *SNCA* were 15% and 26%, while the results were 38% and 52% in smokers with LUAD. However, the methylation status of *CCNA1* was not detected in sputum from smokers with LUAD [70]. In other words, DNA methylation analysis has the potential to distinguish between smokers who need to receive LDCT examinations and who need not to receive LDCT examinations.

### Sputum DNA methylation-based liquid biopsy for the prognosis of LC

As for the prognosis, Belinsky SA et al. conducted a study to evaluate the association between LC recurrence and eight genes methylation status. The eight genes were *CDKN2, MGMT, DAPK1, RASSF1, GATA4, GATA5, PAX5α,* and *PAX5β*. Three indexes, including low (0–1), medium (2–3), and high (≥ 4) methylation index, were used to assess the time to relapse for LC patients via the Kaplan–Meier analysis. However, results showed no significant association between LC recurrence and eight genes methylation. Nevertheless, the authors were interested in whether eight genes methylation status predicts therapeutic response. The association between selenium treatment, risk of recurrence, and gene methylation status was estimated using the generalized estimating equation. The follow-up time lasted for two years. Results revealed that no significant difference of the eight genes methylation levels was observed between LC patients with recurrence after selenium treatment and those without recurrence after selenium treatment [74]. Therefore, the role of DNA methylation in sputum to monitor the prognosis of LC should be studied constantly.

## Bronchoalveolar lavage fluid, bronchial aspirates, and bronchial washings DNA methylation-based liquid biopsy for LC detection

Nowadays, fiberoptic bronchoscopy is a routine examination for patients with suspected LC. Bronchoalveolar lavage fluid is obtained from fiberoptic bronchoscopy examination with minimal invasion [75]. Since bronchoalveolar lavage fluid is anatomically adjacent to tumor cells, it can serve as an alternative source of biomarkers for LC. Usually, specimen from bronchoalveolar lavage fluid is used for histologic or cytologic examination. However, the diagnostic power for LC is limited [76]. Recently, the association between DNA methylation and LC has been studied intensely [77, 78]. Moreover, several studies have reported the status of hypermethylation of gene promoters in the body fluid of LC, including bronchoalveolar lavage fluid (Table 4). Recently, the genes *RASSF1A* and *SHOX2* methylation in bronchoalveolar lavage fluid have been studied extensively. In 2017, Ren M et al. enrolled in 123 LC cases and 130 healthy controls to investigate the relationship between LC and the methylation levels of *RASSF1A* and *SHOX2*. They found that the methylation-positive rates of *RASSF1A* and *SHOX2* were higher in LC group (50.4% and 64.2%) compared to the control group (3.8% and 7.7%). The diagnostic panel of these two genes achieved a sensitivity of 71.5% and a specificity of 90.0%, whereas the sensitivity and specificity of cytological examination were 5.7% and 99.2%, respectively [79]. In the same year, another study enrolled in 284 LC cases and 38 healthy controls for *RASSF1A* and *SHOX2* methylation analysis in bronchoalveolar lavage fluid. At first, they found that compared to healthy controls, the methylation levels of *RASSF1A* and *SHOX2* were significantly higher in LC patients. The sensitivities of *RASSF1A* and *SHOX2* methylation were higher than cytology and serum CEA. Then, a panel of *RASSF1A* and *SHOX2* genes showed an AUC of 0.892, while the AUC of cytology and serum CEA were 0.828 and 0.741, respectively. The panel showed the highest sensitivity of 81% compared to cytology (68.3%) and serum examination (30.6%). The above results illustrated the advantages of *RASSF1A* and *SHOX2* methylation analysis in bronchoalveolar lavage fluid to screen LC over conventional approaches, such as cytology and serum CEA [76]. In 2004, Topaloglu O et al. examined promoter methylation status of 8 genes (*CDH1, AFR, GSTP1, p16, RAR-β2, MGMT, RASSF1A,* and *APC*) using bronchoalveolar lavage fluid from 31 LC patients and 10 age-matched controls. Overall, 21 of 31 LC patients were positive for promoter methylation. For the 10 controls, *MGMT, RAR-β2, AFR, GSTP1,* and *p16* were not observed to be methylated, while methylation levels of the 3 remaining genes were very low [80].

**Table 4:** Bronchoalveolar lavage fluid, bronchial aspirates, and bronchial washings DNA methylation-based liquid biopsy for LC

Purpose	Genes	Cases	Controls	Sensitivity/specificity	Samples	Methods	References
Diagnosis	*RASSF1A/SHOX2*	123	130	71.5/90.0%	BALF	RT-PCR	[79]
Diagnosis	*RASSF1A/SHOX2*	284	38	81.0/97.4%	BALF	RT-PCR	[76]
Diagnosis	*CDH1/AFR/GSTP1/p16/RAR-β2/MGMT/RASSF1A/APC*	31	10	–/–	BALF	RT-PCR and RT-MSP	[80]
Diagnosis	*SHOX2*	281	242	68.0/95.0%	bronchial aspirates	RT-PCR	[81]
Diagnosis	*PHF11/PDGFRA/TFAP2A/PRR15/HOXA11/TOX2/TBX15*	70	53	87.0/83.3%	bronchial washings	450 K Methylation BeadChip and Pyrosequencing	[82]

Besides bronchoalveolar lavage fluid, bronchial aspirates and bronchial washings are both important tools for diagnosing LC. Like bronchoalveolar lavage fluid, they are obtained with little risk and without extra effort during the first fiberoptic bronchoscopy [83]. From an anatomical point of view, they are close to LC cells and tumor microenvironment. Thus, they can reflect molecular changes and provide tools for detecting LC [84] (Table 4). In 2010, a study consisting of 281 LC cases and 242 healthy controls was conducted. The result showed that DNA methylation of *SHOX2* in bronchial aspirates could discriminate LC patients from healthy people with an AUC of 0.86, a sensitivity of 68%, and a specificity of 95%. Furthermore, the performance of *SHOX2* methylation level was perfect for diagnosing small cell lung cancer (SCLC) with a high sensitivity of 97%. For squamous cell carcinoma (SCC), the sensitivity was 82%. However, the sensitivity for adenocarcinoma was lower than other subtypes. Subsequent analysis demonstrated that the *SHOX2* methylation marker could detect a higher stage of LC with a slightly increased sensitivity. The reason may be that there are more malignant tumor cells in bronchial aspirates of advanced LC patients [81]. Another study established a panel based on *PHF11, PDGFRA, TFAP2A, PRR15, HOXA11, TOX2,* and *TBX15* genes to diagnose LC patients. Results showed that the AUC was 0.87 with a sensitivity of 87% and a specificity of 83.3% [82].

## Other DNA methylation-based liquid biopsies for LC

Besides cfDNA, sputum, bronchoalveolar lavage fluid, bronchial aspirates, and bronchial washings, liquid biopsy also includes CTCs, exosomes, urine, pleural effusions, and saliva [13–16]. The currently reported CTCs, exosomes, urine, pleural effusions, and saliva DNA methylation-based liquid biopsy for LC were summarized in Table 5.

**Table 5:** Other DNA methylation-based liquid biopsy for LC

Purpose	Genes	Cases	Controls	Sensitivity/specificity	Samples	Methods	References
Diagnosis	CpG sites	15	5	–/–	CTC	LCM-μWGBS and PCR	[85]
Subtype	CpG sites	884	154	–/–	CTC	450 K Methylation BeadChip and WGBS	[86]
Diagnosis	*SOX17/HOXA9/CDO1/TAC1*	74	27	93.0/30.0%	Urine	qMSP	[87]
Diagnosis	*CDO1/SOX17/TAC1*	23	60	–/–	Urine	qMSP	[88]
Prognosis	*MGMT/BRCA1/RARβ/p16/INK4a*	30	0	–/–	Pleural effusion	MSP	[89]

### CTC DNA methylation-based liquid biopsy for LC

CTCs are tumor cells in cancer patients’ peripheral blood originating from primary or metastatic malignant tumors. CTCs cause tumor metastases, and the number of CTCs in the bloodstream is related to patients’ therapeutic response. CTCs provide information at DNA, RNA, and protein levels. As a type of liquid biopsy, CTCs carry genetic and epigenetic information on tumor cells and CTCs examination is noninvasive [90–93]. Thus, CTCs have the potential to be biomarkers of LC. Previous studies have revealed that CTCs were existed in the blood of LC patients, especially in stage I LC [94–96]. For CTCs DNA methylation of LC, Zhao L et al. found that the methylation levels of cancer tissues were higher than normal tissues. Moreover, the methylation levels of CTCs were lower than both normal and cancer tissues, suggesting a loss of DNA methylation during carcinogenesis of LC from normal tissues to cancer tissues, and to CTCs [85]. Jiang JH et al. tried to use DNA methylation of CTCs to classify the NSCLC subtypes. They found 5426 differentially methylated CpG sites to discriminate between LUAD and lung squamous cell carcinoma (LUSC), 1409 differential methylated CpG sites to discriminate between LUAD and normal tissues, and 2919 differential methylated CpG sites to discriminate between LUSC and normal tissues. The diagnostic accuracies were 97.5%, 95.7%, and 100%, respectively [86].

### Exosome DNA methylation-based liquid biopsy for LC

Exosomes are spherical lipid bilayer vesicles which are secreted by cells. The diameter of the exosome is around 40-100 nm, and the density of exosome is about 1.13–1.19 g mL^−1^ [97, 98]. Many studies have verified that exosomes exist in various body fluids, such as plasma, serum, saliva, tears, urine, semen, amniotic fluid, cerebral spinal fluid, bronchoalveolar lavage fluid, and bile [99–108]. Exosomes contain proteins, nucleic acids, lipids, transcription factor receptors, cytokines, and other biological substances [109, 110]. Therefore, exosomes can serve as biomarkers for LC detection [111–114]. Several pieces of research studied DNA methylation of exosomes in different types of cancer, such as prostate cancer, diffuse large B cell lymphoma, and murine melanoma [115–118]. However, the studies about DNA methylation of exosomes for LC detection were limited, which needs further research.

### Urine DNA methylation-based liquid biopsy for LC

As a kind of liquid biopsy, urine has been a forthcoming method of detection for LC [87, 119, 120]. There are some advantages of urine analysis. The collection of urine is easy and noninvasive. Besides, a large urine volume can be obtained in a one-time collection. Furthermore, DNA in urine is more stable than other body liquids and urine can be stored at room temperature [87, 88]. In 2020, Liu B et al. found that the positive methylation rates of *SOX17, HOXA9, CDO1,* and *TAC1* of urine in LC patients were higher than in healthy controls. For lung cancer risk prediction, univariate logistic regression analysis revealed that methylation levels of *SOX17, HOXA9, CDO1,* and *TAC1* were significantly related to lung cancer risk [87]. In 2021, Bach S et al. analyzed the DNA methylation status of 23 urine samples from NSCLC patients and 60 urine samples from healthy controls. The results showed that methylation levels of *SOX17* and *TAC1* were significantly different between NSCLC patients and healthy controls [88].

### Pleural effusion DNA methylation-based liquid biopsy for LC

Malignant pleural effusion is a usual complication of various malignant tumors, especially LUAD [121–124]. In most cases, the production of malignant pleural effusion occurs in advanced or metastasized cancers [125]. Thus, pleural effusion analysis may provide prognosis information on LC. Previous studies have examined the role of *p16/INK4a, MGMT, BRCA1,* and *RARβ* in the recurrence and development of NSCLC [126]. In 2012, Botana-Rial M et al. analyzed the relationship between DNA methylation of the above four genes and the survival of LUAD patients. Compared to LUAD patients with 1–3 methylated genes, a shorter survival time was found in patients with no methylated genes. Cox multivariate analysis revealed that hypermethylation in one or more genes had been associated with a better prognosis [89].

### Saliva DNA methylation-based liquid biopsy for LC

In recent years, saliva-based liquid biopsy for cancer has received much attention [127–132]. Analyzing components of saliva can reflect genomic and epigenomic alterations involved in physiological and pathological processes, and thus reflecting a person’s physical conditions [133–135]. Previous studies have suggested that some methylation profiles in saliva were similar to those in tissue [135]. Moreover, saliva can be collected in a noninvasive, cost-effective, fast, and reliable way [135, 136]. Therefore, salivary biomarkers can be applied to early detection, prognosis, and therapeutic effect response of cancer. Recently, saliva DNA methylation-based liquid biopsy investigations mainly focused on oral cancer and head and neck cancer [137–139]. However, the study about LC screening based on saliva DNA methylation is limited and needs further explorations.

## DNA methylation profile based on liquid biopsies between smokers and non-smokers for LC

Smoking can alter epigenetic mechanisms, including histone modifications and DNA methylation [140]. Several studies have demonstrated the relationship between DNA methylation and smoking. Here, we focused on liquid biopsy-based DNA methylation profile of smokers and non-smokers. In 2013, Ostrow KL et al compared the *NISCH* gene methylation level in plasma between light smokers (< 20pack/year) and non-smokers. The result revealed that in light smokers, 69% of LC patients showed *NISCH* promoter methylation, and *NISCH* methylation was absent in those without LC. Moreover, methylation alterations of *NISCH* promoter can be induced by smoking before any detectable malignant tumors [141]. Another study reported that compared to former and non-smokers, methylation levels of *F2RL3* and *AHRR* in peripheral blood DNA were lower in current smokers, indicating that smoking may lead to cancer risk [142]. The e-cigarette is becoming increasingly popular and the effects of e-cigarette use on health have attracted researchers’ attention. In 2021, Richmond RC et al. conducted a study, including 116 e-cigarette smokers, 117 cigarette smokers, and 117 non-smokers, to analyze DNA methylation in participants’ saliva. They found that DNA methylation levels at 7 CpGs were related to e-cigarette use. Moreover, the DNA methylation profile of e-cigarette smokers was largely different from cigarette smokers. The diagnostic value for LC detection of DNA methylation profile related to e-cigarette use was worse than that of cigarette use, which requires further investigations [143].

## Discussion

Enhancing the early diagnosis rate of LC is clinically critical for improving its prognosis. However, early detection of LC is still difficult because of the high false-positive rate of LDCT, the low specificity of conventional serum tumor markers, and the traumatic nature of aspiration biopsy. Liquid biopsy has the advantages of minimal invasiveness and the ability to monitor tumor dynamically [144]. Previous studies have demonstrated that aberrant DNA methylation, such as hypermethylation in tumor suppressor genes or hypomethylation in oncogenes, is an early event in tumorigenesis [145, 146]. Thus, DNA methylation-based liquid biopsy opens up new pathways for the early detection of LC. As the concentration of DNA may be very low under some circumstances, how to detect DNA methylation status accurately and quickly is of great importance, which has attracted the attention of multiple researches [147–150]. With the rapid development of technology, DNA methylation detection will become increasingly efficient. In this review, we first summarized currently used detection approaches focusing on genome-wide and locus-specific DNA methylation analysis. Then, a series of newly developed methods were also introduced, such as mass spectrometry and biosensors. Those studies provide insight into areas which could improve the detection efficiency and help discover cost-effective and clinically practical methods.

In the following, we reviewed different types of liquid biopsies for the detection and prognosis of LC. CfDNA is a typical sample, as tumor cells release DNA into peripheral blood and a patient is very receptive to blood drawing. CfDNA methylation-based liquid biopsy is of great value to the early detection and prognosis of LC. As ctDNA is derived from primary or metastatic tumor cells, most of the studies focused on ctDNA methylation status of LC. Plasma-derived ctDNA is more stable than serum-derived one, so most of the samples are serum. Compared to the single gene, the diagnostic efficiency of multiple genes panel is more powerful. Another important sample of LC patient is sputum, which is absolutely noninvasive and easy to be obtained. Not all smokers eventually develop LC. Moreover, LDCT has potential radioactive harms to patients [151]. Fortunately, DNA methylation analysis of sputum has the potential to select smokers at high risk for developing LC and recommend them to receive LDCT scans. Particularly, the value of multiple genes panel is higher than a single gene. Although Belinsky SA et al. performed a study to use DNA methylation status of sputum to evaluate LC prognosis, they found that LC recurrence and selenium therapeutic response were not related to DNA methylation levels. Further investigations should be conducted to resolve this issue. As fiberoptic bronchoscopy is a routine examination for patients with suspected LC, bronchoalveolar lavage fluid is easy to be obtained from fiberoptic bronchoscopy examination with minimal invasion. The value of DNA methylation-based bronchoalveolar lavage fluid for LC focused on early diagnosis. Remarkably, the gene *RASSF1A* has powerful diagnostic value for LC in several samples, such as bronchoalveolar lavage fluid, sputum, and cfDNA. Also, the gene *SOX17* in cfDNA, sputum, and urine is of value for LC detection and prognosis. Nevertheless, studies about CTC, exosome, urine, pleural effusion, and saliva-based DNA methylation analysis of LC are limited, which need to be further explored.

## Conclusions and future perspectives

**Fig. 3:**
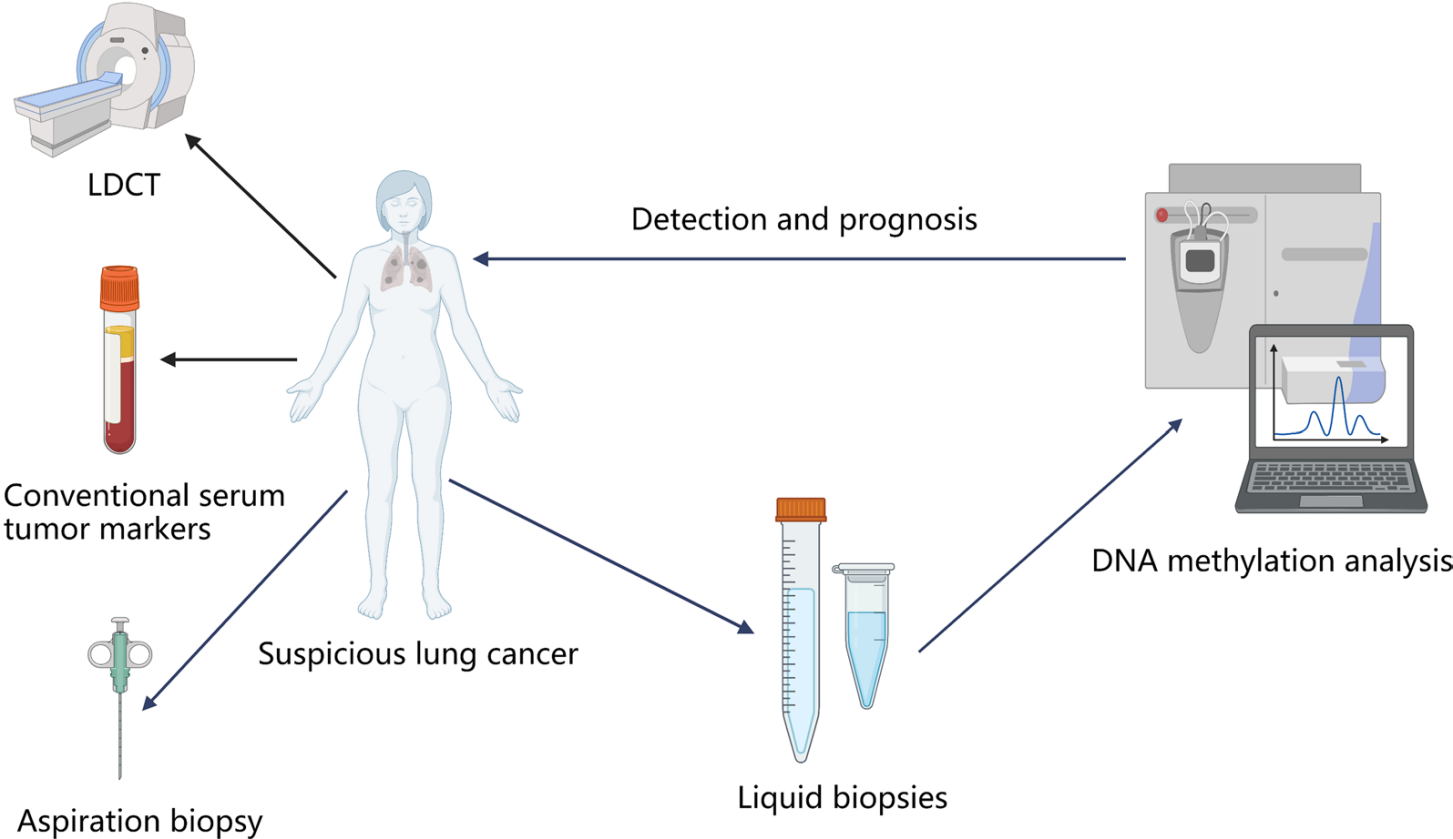
The sketch map of DNA methylation-based liquid biopsies for the detection and prognosis of LC, which are superior to LDCT, conventional serum tumor markers, and aspiration biopsy

Our review systematically summarized the latest research on DNA methylation based on liquid biopsies for early diagnosis and prognosis assessment of LC. Based on this review, DNA methylation-based liquid biopsy has a promising future as a novel biomarker for LC detection and prognosis (Fig. 3). As a noninvasive approach, liquid biopsy has great potential to be the biomarkers for the early detection, recurrence monitoring, and prognosis of cancer. We guess that when coupled to LDCT and serum tumor markers, DNA methylation-based liquid biopsy may have a better diagnostic performance. Nevertheless, liquid biopsy still requires standardization of specimen collection, DNA isolation, and DNA quantification. Furthermore, we need to improve its clinical application. Compared to other liquid biopsies, exosomes and saliva-based DNA methylation analysis of LC is still limited, which requires further research to fill the gaps. Considering the fact that sometimes the concentration of DNA in body liquid may be very low, we need to explore highly sensitive detection technologies and tools to overcome the difficulty.

## Data Availability

Not applicable.

## Abbreviations

LC: Lung cancer
LDCT: Low-dose computed tomography
cfDNA: Cell-free DNA
DNMTs: DNA methyltransferases
SAM: S-adenosylmethionine
TFs: Transcription factors
CTCs: Circulating tumor cells
NSCLC: Non-small cell lung adenocarcinoma
TKIs: Tyrosine kinase inhibitors
ctDNA: Circulating tumor DNA
ELISA: Enzyme-linked immunosorbent assay
LUMA: Luminometric methylation assay
NGS: Next-generation sequencing
MeDIP: Methylated DNA immunoprecipitation
MBD: Methyl-CpG binding domain
MethylCap: Methyl-CpG binding domain protein capture
HELP: HpaII tiny fragment enrichment by ligation-mediated PCR
WGBS: Whole-genome bisulfite sequencing
RRBS: Reduced representation bisulfite sequencing
MSP: Methylation-specific PCR
dPCR: Digital PCR
ddPCR: Droplet digital PCR
OS: Overall survival
PFS: Progression-free survival
gDNA: Genomic DNA
NLST: National lung screening trial
SCLC: Small cell lung cancer
SCC: Squamous cell carcinoma
LUSC: Lung squamous cell carcinoma
